# Endometrial Cancer Cells Promote M2-Like Macrophage Polarization by Delivering Exosomal miRNA-21 under Hypoxia Condition

**DOI:** 10.1155/2020/9731049

**Published:** 2020-10-13

**Authors:** Li Xiao, Yumin He, Fan Peng, Jianlin Yang, Chengfu Yuan

**Affiliations:** ^1^Medical College of China Three Gorges University, China; ^2^China Three Gorges University Hubei Key Laboratory of Tumor Microenvironment and Immunotherapy, China

## Abstract

Increasing evidence has demonstrated that hypoxia was an aggressive feature in endometrial cancer (EC), which is significantly associated with the tumor grade, lymph node metastasis, and tumor resistance to chemotherapy. However, the relationship between hypoxia and the immune microenvironment in EC is not very clear. Exosomes are small membrane vesicles secreted from a variety of cell types which mediate cell-to-cell communication through transported biomolecules. Here, we investigated whether exosomes can play an immunomodulatory role in intercellular communication between EC cells and macrophages. EC KEL cells were cultured under hypoxia or normoxic condition to collect exosomes. After identification, the exosomes derived from hypoxic or normoxic KEL cells were cultured with the monocyte cell line THP-1 to study the immunoregulation function of KEL cells. The results showed that the total number of exosomes produced by hypoxic KEL cells was significantly higher than that in normoxic condition. In addition, hypoxia markedly stimulated the increase in miRNA-21 expression in the exosomes. After coculture, we found that exosomal miRNA-21 could be horizontally transferred into THP-1 cells. And then, the notably enhanced mRNA expression levels of IL-10 and CD206 in THP-1 cells were observed, suggestive of M2 polarization. To further study the effect of miRNA-21-containing exosomes, we transfected miRNA-21 mimics or inhibitor into THP-1 cells. The results showed that miRNA-21 mimics promoted IL-10 and CD206 mRNA expression levels, and the miRNA-21 inhibitor significantly prevented the alteration induced by intake of hypoxic KEL cell-derived exosomes. In summary, we found that endometrial cancer KEL cells in hypoxic condition promoted monocyte THP-1 cell transformation to M2-like polarization macrophages through delivering exosomal miRNA-21, which may be a potential mechanism of the formation of the immune microenvironment in EC progression.

## 1. Introduction

Endometrial cancer (EC) is the most common malignancy of the female genital tract and the sixth most frequent cancer among women in the world. The incidence of EC and the EC-related mortality rate are increasing during recent years, especially in western countries. Only in the United States, 1,762,450 new cases and 606,880 cancer-related deaths are estimated to occur in 2019 [1]. Standard methods of EC treatment include surgery followed by radiotherapy and chemotherapy. Despite the rapid development of diagnosis and treatment, the prognosis of patients with advanced and recurrent EC is still poor [2]. Thus, additional exploration on the mechanism of the occurrence, development, proliferation, and metastasis of EC to define effective intervention strategies against EC progression is urgently needed.

With the deepening of research, it has been proven that tumorigenesis and development are affected by the tumor microenvironment in which tumor-associated macrophages (TAMs) are the most important research topic [3]. According to their functions, TAMs tend to acquire a polarized M2 phenotype in many kinds of tumors which was related to the poor prognosis of a tumor through various mechanisms [4, 5]. Recently, several studies also have indicated an inverse correlation between M2 TAM infiltration and EC prognosis. The high density of M2-like TAMs was related to lymph node metastasis, higher histological grade, and increased expression of Ki-67 and p53 in EC [6]. CD68+CD163+ macrophage infiltration in EC was significantly higher than that in normal endometrial tissues, which is positively correlated with the development of EC by increasing the sensitivity of the tumor microenvironment to estrogen [7]. TAM-derived CXCL8 mediated the downregulation of ER*α* expression of EC cells via HOXB13, which may be associated with cancer invasion, metastasis, and poor prognosis [8]. However, the mechanism of M2-like TAM differentiation in EC is still unclear.

In this study, we observed the effect of endometrial cancer cells on macrophages and explored the potential mechanism of the formation of M2-likeTAM cells. We showed that the total number of exosomes produced by EC KEL cells was significantly increased in a hypoxic environment. And more, the exosomes carried higher levels of miRNA-21 derived from KEL cells in a hypoxia environment, compared with normoxic condition. These exosome were horizontally transferred into monocytes and promoted monocyte transformation to M2-like polarization macrophages through delivering miRNA-21, which may be an underlying mechanism of immune escape in the tumor microenvironment.

## 2. Material and Methods

### 2.1. Cell Culture

The endometrial cancer cell line KEL was incubated in a humidified atmosphere at 37°C and 5% CO_2_ in RPMI1640 medium (Gibco) supplemented with 100 U/mL penicillin, 100 mg/mL streptomycin, and 10% exosome-depleted FBS (System Biosciences). For the induction of stress conditions, e.g., hypoxia, KEL cells were cultured in a hypoxic chamber (<3% CO_2_) or under standard conditions in parallel serving as a normoxia control. Following the treatment, the supernatant was collected and immediately analyzed. All experiments were performed in triplicate.

In order to study the immunoregulation of KEL cells, the monocyte cell line THP-1 was cocultured with exosomes (28 ng/mL) derived from KEL cells under hypoxia or normoxia condition for 24 h. Then, the cells were collected to detect the mRNA expression of cytokines. The culture media were harvested to assay the protein expression level of cytokines by ELISA.

### 2.2. Exosome Isolation and Characterization

A total of 10 mL of culture media was mixed with ExoQuick exosome precipitation solution, and exosome isolation was performed according to the manufacturer's instructions (ExoQuick Exosome Isolation and RNA Purification Kit, System Biosciences (SBI)).

Exosomes were characterized by transmission electron microscopy, nanoparticle tracking analysis (NTA), and western blot in terms of size, concentration, and surface marker. The concentration and size distribution of exosomes were analyzed with the NanoSight NS300 system (NanoSight, Amesbury, UK). Each sample was measured three times.

### 2.3. ELISA

The cell-free culture supernatant was harvested and assayed by ELISA for TNF-*α*, IL-1*β*, and IL-10 according to the manufacturer's protocols. The detection limits were as follows: TNF-*α* (BD Bioscience PharMingen), 7.8 pg/mL; IL-1*β* (Invitrogen), 2 pg/mL; and IL-10 (Invitrogen), 2 pg/mL. For convenient analysis, all values of less than the detectable limit were considered zero.

### 2.4. Immunofluorescence Assay

To observe the uptake of exosomes by THP-1, the purified exosomes derived from KEL cells were labeled with the red fluorescent linker PKH26 (Sigma) and treated with THP-1 cells in the final concentration of 28 ng/mL for 12 h. Afterwards, a cell smear was prepared and the cover slips were mounted onto slides with Fluoroshield mounting medium and DAPI (SouthernBiotech, USA, 1 : 1000). The slides were observed with an Olympus BX53 microscope, and the images were collected with the Cell Sense software (Olympus, Center Valley, PA).

### 2.5. RNA Isolation and RT-PCR Analysis

Both cells and exosomes were lysed, and total RNAs were isolated using the exoRNeasy Serum/Plasma Maxi Kit (Qiagen, Maryland, USA) according to the manufacturer's standard protocol. The extracted RNA was dissolved with 25–40 *μ*L of RNase-free water. The quantity and quality of the RNA were determined by using NanoDrop 1000 (260/280 ratios).

For mRNA analyses, reverse transcription was performed using the GeneAmp RNA PCR Kit (Applied Biosystems, Foster City, CA) and the cDNA was amplified in 96-well reaction plates with a SYBR Green PCR Master Mix (Applied Biosystems) on an ABI 7500 real-time PCR thermocycler. The expression of GAPDH mRNA or U6 was used for standardization, and the relative mRNA expression was calculated by the 2^-*ΔΔ*t^ method.

### 2.6. Cell Transfection

In order to further determine the immunomodulatory effect of exosomal miRNA-21 on THP-1, miRNA-21 mimics or inhibitor was transfected into THP-1 cells in vitro. THP-1 cells were cultured with serum-free medium and were transfected either with miRNA-21 mimics (mature sequence 5′-UAGCUUAUCAGACUGAUGUUGA-3′), miRNA-21 inhibitor (5′-UAGCUUAUCAGACUGAUGUUGA-3′), or miRNA negative control (Sigma-Aldrich) using Lipofectamine 2000 (Invitrogen, Carlsbad, CA) following the manufacturer's instructions. Then, THP-1 cells were cultured in RPMI1640 medium (Gibco) supplemented with 100 U/mL penicillin, 100 mg/mL streptomycin, and 10% exosome-depleted FBS (System Biosciences) at 37°C and 5% CO_2_. In the miRNA-21 inhibitor group, exosomes derived from hypoxic KEL cells with a final concentration of 28 ng/mL were added to the culture medium. After 24 h, the culture medium and cells were collected and cytokine expression was analyzed.

### 2.7. Statistical Analysis

Data are expressed as mean ± SEM or median (range). Comparison between two groups was performed by Student's *t*-tests. Values of *P* < 0.05 (two tailed) were considered significant.

## 3. Results

### 3.1. Endometrial Cancer KEL Cells Released Exosomes in Response to Hypoxia Condition

**Figure 1:**
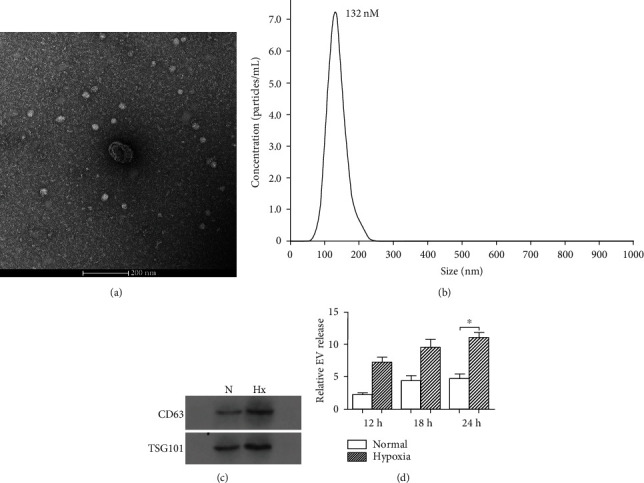
Characterization and quantification of exosomes produced by KEL cells under hypoxia or normoxic condition. KEL cells were incubated under hypoxic or normoxic condition for 12 h, 18 h, or 24 h to collect exosomes. (a) Representative electron micrograph of exosomes isolated from hypoxia- or normoxia-conditioned medium of KEL cells. Scale bar, 200 nm. (b) A representative plot showing the size distribution of exosomes was carried out using nanoparticle tracking analysis (NAT). (c) Western blot analysis showing the presence of CD63 and TSG101 in exosomes derived from KEL cells. (d) Quantification of exosomes produced by KEL cells under hypoxia or normoxic condition at various time points (12 h, 18 h, and 24 h). The measurements were done using the NTA system in triplicate, and data presented as fold change of the EV number. N: normoxic; Hx: hypoxic. ^∗^*P* < 0.05.

Endometrial cancer KEL cells were cultured under hypoxic or normoxic condition, and the culture supernatant was collected to isolate exosomes. Transmission electron microscopy, NTA, and immunoblot analysis were used to confirm morphology, size, and marker protein for the exosome. Electron micrographs revealed that the collected products had a distinctive cup shape (Figure 1(a)). The majority of the particles ranged in size from 90 to 150 nm, indicating an abundance of exosomes (Figure 1(b)). The presence of CD63 and TGT101, which are typical marker proteins of exosomes, was identified by western blotting (Figure 1(c)). These results confirmed that KEL cells released exosomes. And more, the total numbers of exosomes derived from KEL cells were more significantly enhanced under hypoxic than under normoxic condition (*P* < 0.05, Figure 1(d)), suggesting that KEL cells secreted more exosomes in response to the hypoxia microenvironment.

### 3.2. Exosome Derived from KEL Cells under Hypoxia Condition Had Different Characteristics of miRNA Expression

**Figure 2:**
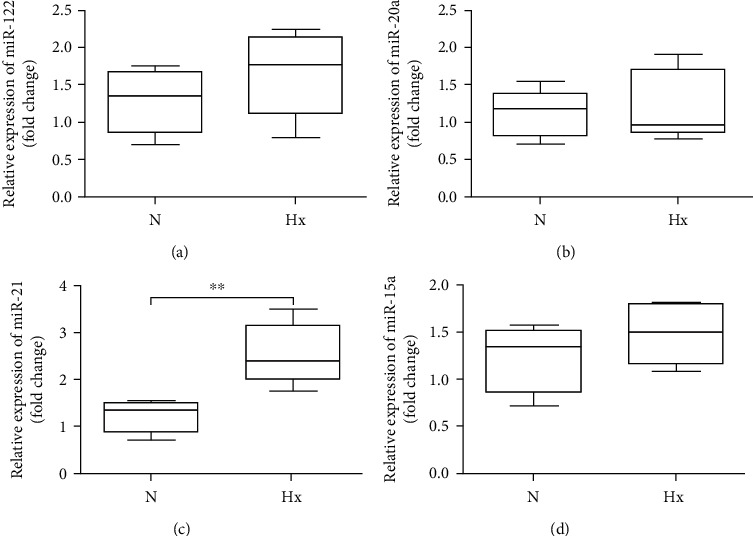
Expression levels of exosome-related miRNA produced by KEL cells under hypoxia or normoxic condition. The expression levels of exosome-related miRNA were detected by RT-PCR. The statistical assay of (a) miR-122, (b) miR-20a, (c) miR-21, and (d) miR-15a contained in exosomes. N: normoxia; Hx: hypoxia. ^∗∗^*P* < 0.01.

Recent work has proven the alterations in miRNA expression patterns in EC cells when exposed to the microenvironmental stress, like hypoxia or acidosis [9]. We detected several kinds of exosome-associated miRNAs secreted by KEL cells which changed under hypoxia condition, such as miRNA-122, miRNA-20a, miRNA-21, and miRNA-15a. The results showed that miRNA-21 expression level in exosomes derived from KEL cells was markedly increased under hypoxic condition, compared to normoxic condition (Figure 2(c)), and no notable change in the expression of miRNA-122, miRNA-20a, and miRNA-15a was found (Figures 2(a), 2(b), and 2(d)).

### 3.3. KEL Cells Transferred miRNA-21 into Monocytes through the Exosome under Hypoxia Condition

**Figure 3:**
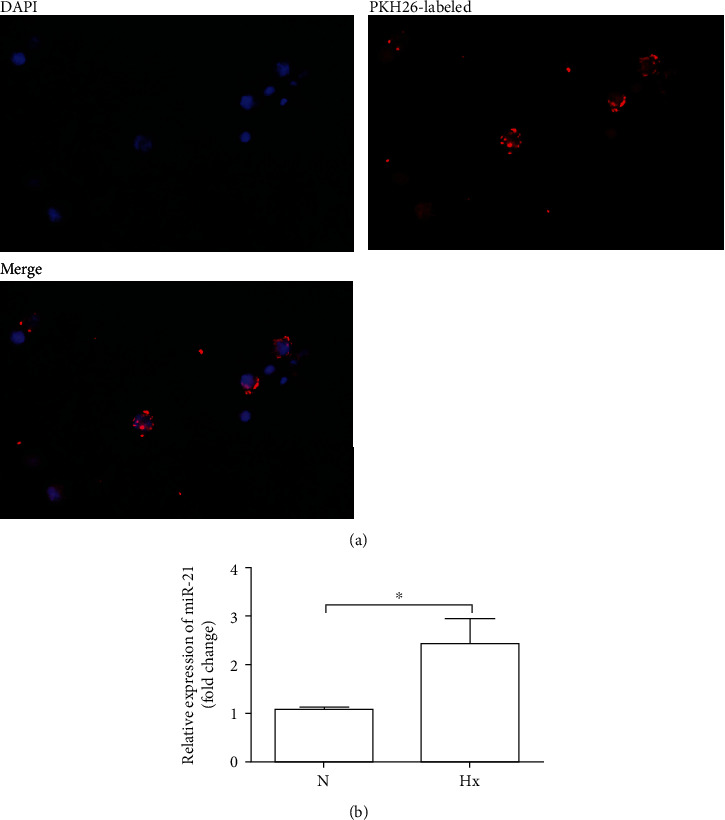
Hypoxic KEL cell-derived exosomes horizontally transferred miRNA-21 to the THP-1 cells. THP-1 cells were treated with exosomes labeled with PKH26 for 12 h, and after that, exosomes were washed off and media replaced. Nuclei were stained with DAPI. miRNA-21 levels were identified in THP-1 cells using RT-PCR. U6 was used to normalize the Ct values between the samples. (a) Representative fluorescence microscopy of THP-1 after incubation with exosomes. (b) Statistical assay of the expression level of miRNA-21 in THP-1 after the uptake of exosomes. N: normoxia; Hx: hypoxia.

To investigate possible crosstalk between KEL cells and immune cells, we labeled exosomes derived from hypoxia-treated KEL cells with red fluorescent PKH26 and cocultured them with human THP-1 monocytes. After a 12-hour coculture of exosomes with THP-1 monocytes, microscopic observation showed that exosomes were taken up by THP-1 cells (Figure 3(a)). More than that, we found only a low copy number of baseline miRNA-21 in monocytes generally. After coculture with exosomes, there was a 2–3-fold increase in the level of miRNA-21 in THP-1. It indicated that exosomes mediated the horizontal transfer of their miRNA-21 cargo into the THP-1 cells (Figure 3(b)).

### 3.4. Exosome Derived from Hypoxic KEL Cells Mediated Monocyte Differentiation and Polarization

**Figure 4:**
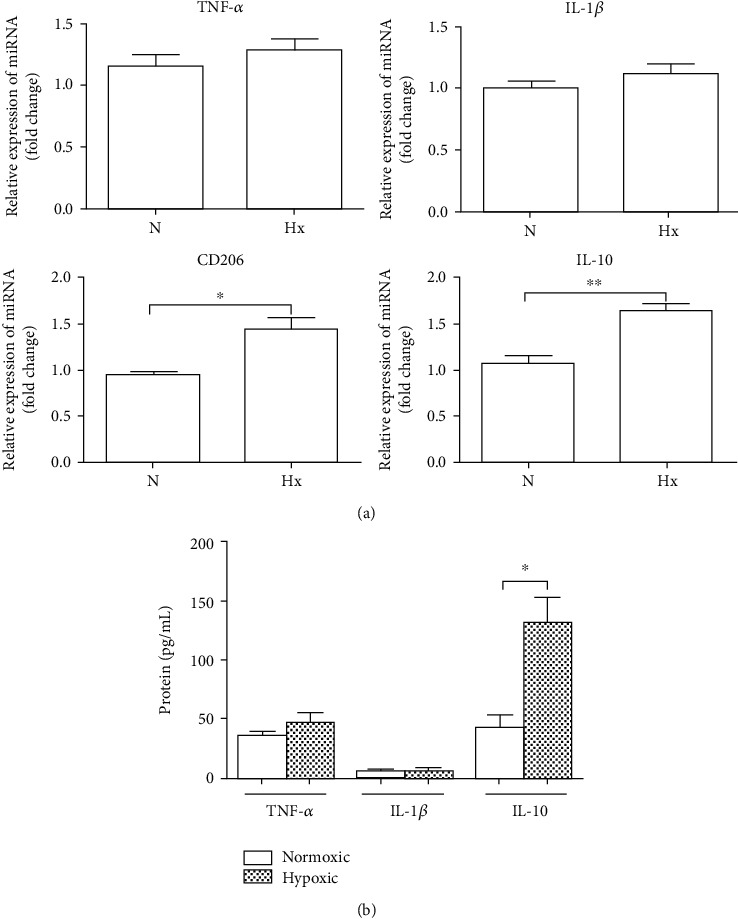
Immunomodulatory effect of exosomes produced by KEL cells on human THP-1 monocytes under hypoxia condition. THP-1 cells were cocultured with exosomes excreted by KEL cells under hypoxia or normoxic condition for 24 h. Then, mRNA or protein expression levels of TNF-*α*, IL-1*β*, IL-10, and CD206 were detected. (a) The statistical assay of mRNA expression of TNF-*α*, IL-1*β*, IL-10, and CD206 in THP-1 cells. (b) ELISA assayed the protein levels of TNF-*α*, IL-1*β*, and IL-10 in the supernatants. The results represent three independent experiments. N: normoxia; Hx: hypoxia. ^∗^*P* < 0.05, ^∗∗^*P* < 0.01.

We therefore observed the immunomodulatory effect of KEL cell-derived exosomes on macrophages. THP-1 cells were cultured in the presence of exosomes excreted by KEL cells for 24 h. Then, mRNA or protein levels of TNF-*α*, IL-1*β*, IL-10, and CD206 in THP-1 cells were analyzed. We found that the mRNA expression levels of IL-10 and CD206 were significantly increased in THP-1 cells treated with exosomes derived from hypoxic KEL cells, compared with normoxic KEL cells (*P* < 0.05, Figure 4(a)). However, no marked change in mRNA expression of TNF-*α* and IL-1*β* in THP-1 cells was found. These suggested the polarization direction of M2 macrophages. Additionally, protein levels of cytokines TNF-*α*, IL-1*β*, and IL-10 were quantified using ELISA, which showed a significant release of IL-10, not TNF-*α* and IL-1*β* (*P* < 0.05, Figure 4(b)).

### 3.5. KEL Cells Promote M2-Like Macrophage Polarization by Delivering Exosomal miRNA-21 under Hypoxia Condition

**Figure 5:**
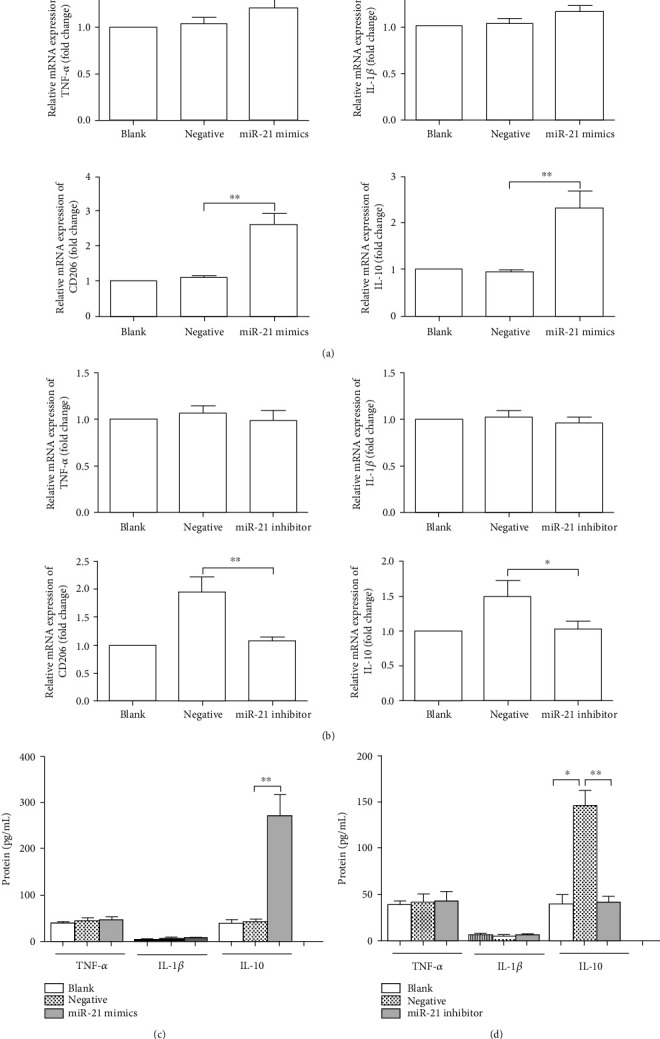
KLE-cell derived exosomal miRNA-21 promoted M2-like polarization of THP-1 monocytes under hypoxia condition. (a) miRNA-21 mimics or negative control was transfected into THP-1 cells for 24 h. The statistical assay of TNF-*α*, IL-1*β*, IL-10, and CD206 mRNA expression in THP-1 cells. (b) miRNA-21 inhibitor and negative control were introduced to the THP-1 cells and then cocultured with exosomes derived from hypoxic KEL cells. The statistical assay of TNF-*α*, IL-1*β*, IL-10, and CD206 mRNA expression in THP-1 cells. (c) The experiment is the same as (a). The statistical assay of the protein levels of TNF-*α*, IL-1*β*, and IL-10. (d) The experiment is the same as (b). The statistical assay of the protein levels of TNF-*α*, IL-1*β*, and IL-10 in the supernatants. The results represent three independent experiments. ^∗^*P* < 0.05, ^∗∗^*P* < 0.01.

To evaluate the functional role of exosomes carrying miRNA-21 in monocyte differentiation and polarization, THP-1 cells were transfected with miRNA-21 mimics, miRNA-21 inhibitor, or negative control in the coculture with hypoxic KEL cell-derived exosomes or not in vitro. Following transfection with miRNA-21 mimics, IL-10 and CD206 mRNA expression levels in THP-1 cells were markedly increased compared with those in negative control-treated THP-1 cells (*P* < 0.01, Figure 5(a)). When THP-1 cells were transfected with the miRNA-21 inhibitor, the increase in IL-10 and CD206 mRNA expression levels induced by the intake of hypoxic KEL cell-derived exosomes was significantly prevented (*P* < 0.05, Figure 5(b)). Similar results were found in protein expression levels of cytokines TNF-*α*, IL-1*β*, and IL-10 (Figures 5(c) and 5(d)). These results suggested that miRNA-21 transferred by EC cell-derived exosomes is functional and modulated the transformation of monocytes into M2 macrophages under hypoxic condition.

## 4. Discussion

As known, hypoxia is common alteration in solid tumors owing to the imbalance between extreme energy demands of rapid cell division and their blood supply. In order to survive and proliferate in a hypoxic microenvironment, tumor cells undergo a series of adaptive changes which are involved in more malignant phenotypes like angiogenesis, metastasis, and tumor resistance to chemotherapy or radiotherapy [10]. Similar to other solid tumors, hypoxia is an aggressive feature in EC. Hypoxic condition reduced the expression of HtrA3 which in turn promotes EC progression [11]. HIF-1*α*, a master regulator of cell adaptation to hypoxia, was overexpressed in the vast majority of EC cases [12, 13], especially in postradiation recurrences [14]. Increasing evidence has demonstrated that HIF-1*α* promoted the progression of EC through various mechanisms and was significantly associated with the tumor grade, lymph node metastasis, and tumor resistance to chemotherapy [15, 16]. However, the relationship between hypoxia and the immune microenvironment in EC is not very clear. In this study, we tried to elucidate the partly mechanism of hypoxia affecting the formation of the immune microenvironment in EC.

Exosomes are small vesicles ranging from 30 to 150 nm produced by almost all cell types and mediate cell-cell crosstalk in cancer tissue through containing biomolecules [17, 18]. Previous studies have shown that the total amount of microparticles in EC patients was much higher than that in healthy controls, which correlated with the histologic grade and clinical stage of the tumor [19]. Exosomes derived from EC cells or cancer-associated fibroblasts were transported into neighbor cells to mediate intercellular communication through the alteration of microRNAs and promoted EC invasion and metastasis [20]. The differential miRNA and circRNAs in exosomes derived from serum [21], urine [22], or peritoneal lavage [23] were confirmed and can be utilized for the discovery of noninvasive biomarker signatures in EC diagnosis. Increasing research showed that tumor cells excreted the altered phenotypic characteristics of exosomes in order to cope with hypoxia [24]. Hypoxia enhances exosome-mediated shuttling of lncRNAUCA1 into bladder cancer cells to promote tumor growth and progression in vitro and in vivo [25]. Hypoxic lung cancer-secreted exosomal miR-23a are potent inducers of angiogenesis and vascular permeability of endothelial cells [26]. Hypoxia-stimulated glioma cells influenced the differentiation and activation of MDSCs through exosomal miRNA-21 [27]. The exosomes secreted by EC cells in the hypoxia environment are rarely reported. In the study, we applied hypoxia condition to KEL cells in order to collect exosomes in the culture medium. Through the analysis of exosomes' size, concentration, and surface marker, the results showed that EC cells excreted higher levels of exosomes under hypoxia condition which were rich in miRNA-21, compared with normoxia condition.

MicroRNA-21 is one of the most frequently upregulated microRNAs in malignant tumors. Expression of miR-21 was found to be significantly upregulated in the EC sample compared with normal, atypical hyperplasia, or benign lesion of the endometrium [28]. The meta-analysis demonstrated that serum miR-21 could serve as a novel biomarker for EC [29]. The overexpression of miRNA-21 modulated EC cell proliferation through the downregulation of PTEN [30] and strongly augments L1CAM gene expression to promote EC cell invasion and metastasis formation [31]. And more, recent work has proven that application of hypoxia resulted in the upregulation of miR-21 in the EC cell line EFE-184 in vitro [9]. Similar to the report, we found that the expression level of miRNA-21 was increased in KEL cells under hypoxic condition, compared with normoxic condition (data not shown). Importantly, exosomes excreted by hypoxic KEL cells contained higher levels of miRNA-21 than those excreted by normoxic KEL cells, which could be transferred into monocytes. It has been reported that the induction of miR-21 positively regulated M2 marker expression of macrophages [32]. So we observed the alteration of monocyte differentiation types. After the uptake of exosomes derived from hypoxic KEL cells, THP-1 cells showed a significant increase in the expression of IL-10 and CD206, which suggested the transformation of M2-like macrophage polarization. To further study the role of miRNA-21 in THP-1 cells, we transfected miRNA-21 mimics or inhibitor into THP-1 cells. The results showed that miRNA-21 mimics promoted the polarization of THP-1 cells to M2-like macrophages and the miRNA-21 inhibitor significantly prevented the polarization induced by the intake of hypoxic KEL cell-derived exosomes.

## Data Availability

The data that support the findings of this study are available from the corresponding author upon reasonable request.
